# Succinate Accumulation Accelerates Oxidative Stress to Promote Pulmonary Epithelial Cell Apoptosis During Lung Ischemia–Reperfusion Injury

**DOI:** 10.1111/jcmm.70645

**Published:** 2025-06-08

**Authors:** Wenhao Wang, Nana Feng, Qi Shi, Jichun Yang, Yulong Tan, Wenyong Zhou, Meng Shi

**Affiliations:** ^1^ Department of Thoracic and Cardiovascular Surgery Huashan Hospital, Affiliated With Fudan University Shanghai China; ^2^ Department of Respiratory and Critical Medicine Shanghai Eighth People's Hospital Shanghai China; ^3^ Department of Clinical Medicine Shanghai Medical College, Fudan University Shanghai China; ^4^ School of Integrative Medicine Tianjin University of Traditional Chinese Medicine Tianjin China; ^5^ Department of Thoracic Surgery Shanghai Chest Hospital, Shanghai Jiao Tong University School of Medicine Shanghai China

**Keywords:** apoptosis, LIRI, mitochondria, oxidative stress, succinate

## Abstract

During ischemia, succinate accumulates and leads to significant damage to the tissues. The specific role of succinate in lung ischemia–reperfusion injury (LIRI) remains unresolved. Differential metabolites in LIRI were identified through untargeted metabolomics using gas chromatography–mass spectrometry (GC–MS). Type II alveolar epithelial cells (AECs) were cultured and subjected to hypoxia/reoxygenation (H/R) in vitro, while an in vivo LIRI model was developed using C57BL/6 mice. Cytokine levels, lung oedema, histopathological alterations and lung functionality were evaluated. Protein levels were analysed through Western blotting. The mitochondrial membrane potential (Δψm) was measured using the JC‐1 fluorescent dye, and mitochondrial morphology in Type II AECs following H/R damage was observed with a transmission electron microscope (TEM). Oxidative stress and apoptosis markers were detected in lung tissues and Type II AECs. Succinate was increased in the peripheral serum of LIRI patients and the C57BL/6 mices model. Succinate pre‐treatment promotes Type II AEC cell apoptosis and oxidative stress, inhibits mitochondrial membrane potential and damages the alveolar epithelial cells' mitochondrial activity after H/R. Meanwhile, succinate may considerably reduce the amounts of acyl‐CoA oxidase 1 (ACOX1) and isocitrate dehydrogenase 2 (IDH2) protein expression. Importantly, N‐acetyl‐L‐cysteine (NAC) was observed to dramatically retard succinate‐induced cell apoptosis, mitochondrial dysfunction and ROS levels in alveolar epithelial cells following H/R in vivo, with succinate‐neutralising antibodies protecting LIRI in vitro. In conclusion, during ischemia, the build‐up of succinate contributes to the advancement of LIRI by enhancing mitochondrial oxidative stress and promoting cell apoptosis, and blocking succinate may be a potential target for LIRI treatment.

## 1 Introduction

Temporary disruption of an organ's blood supply results in ischemia–reperfusion (I/R) injury, which damages tissue upon restoration. Lung ischemia–reperfusion injury (LIRI), a severe and life‐threatening condition, may occur in several clinical settings, including single‐lung ventilation, pulmonary embolism, lung transplantation, high‐volume resuscitation and cardiopulmonary bypass during heart surgery [1, 2, 3]. LIRI is a major contributor to high rates of morbidity, mortality, longer hospital stays and high healthcare costs [4]. Despite its clinical significance, efficacious treatments for LIRI are still limited. Therefore, developing innovative drugs or therapies that alleviate and manage LIRI is a fundamental objective [5]. Consequently, exploring novel treatment approaches for managing LIRI is crucial.

Lung cells are especially vulnerable to oxidative stress because of the physiological conditions of the lung, which include a sufficient blood supply and extended exposure to high oxygen levels [6]. Reactive oxygen species (ROS) are principal agents of oxidative stress. Under normal conditions, ROS helps maintain cellular homeostasis by regulating specific signalling pathways [7]. The main endogenous source of ROS is mitochondria. Excessive formation of ROS that exceeds the cell's antioxidant capacity results in mitochondrial dysfunction, an essential component in both bioenergetic and non‐bioenergetic pathological pathways associated with multiple lung diseases [8]. During I/R episodes, mitochondria generate excessive ROS, triggering a cascade of cellular damage. This includes mitochondrial membrane depolarization, disruption of Ca^2+^ homeostasis, mitochondrial lipid peroxidation and DNA damage. Following these processes, cytochrome c is released into the cytoplasm, initiating apoptotic pathways [9, 10, 11]. Thus, mitochondrial activity is a crucial factor in determining cell survival following ischaemia damage.

Recent studies have underscored the pivotal role of mitochondria in LIRI, revealing that mitochondrial dysfunction can intensify oxidative stress and worsen pulmonary tissue damage [12, 13]. Specifically, the opening of mitochondrial permeability transition pores (mPTP), excessive production of ROS and mitochondrial membrane depolarization have been implicated in enhancing apoptotic signalling and inflammatory responses during LIRI [6]. Moreover, mitochondrial fragmentation and compromised oxidative phosphorylation are associated with the deterioration of pulmonary endothelial and epithelial cell functions, leading to increased vascular permeability and pulmonary edema [14]. These insights suggest that addressing mitochondrial dysfunction could offer a promising therapeutic strategy to alleviate LIRI‐induced damage. There is also strong evidence that the pathophysiology of renal IR damage is influenced by mitochondrial dysfunction, which is caused by an excessive buildup of ROS PEVuZE5vdGU [15, 16]. Accumulated ROS disrupts mitochondrial dynamics by causing abnormal and excessive fragmentation, triggering strong pro‐inflammatory responses that produce mitochondrial dysfunction and contribute to lung damage [17].

Being a methylated derivative of succinic acid, 2,2‐dimethylsuccinic acid has been an essential metabolic step in the tricarboxylic acid (TCA) cycle [18]. Recent studies have shown succinate buildup as a significant metabolic signature and pathological marker associated with increased ROS generation during IR injury [19, 20]. This highlights the need to decrease oxidative stress to prevent lung damage caused by hypoxia‐reoxygenation (H/R). While succinate itself plays a key role in oxidative stress, our study also identified 2,2‐dimethyl succinate as a relevant metabolite under I/R injury. Although 2,2‐dimethyl succinate is not a direct precursor of succinate in the TCA cycle, it was selected due to its significant role in the metabolic changes observed in both animal and clinical models. 2,2‐Dimethyl succinate has previously been identified as a marker linked to oxidative stress and mitochondrial dysfunction, which are key features of LIRI [19]. By focusing on this metabolite, we sought to explore the broader metabolic consequences of I/R, distinct from the direct involvement of succinate, thus offering a more comprehensive understanding of LIRI's pathophysiology. In particular, we explore how succinate accumulation interacts with mitochondrial dysfunction in type II pneumocytes during H/R, providing novel insights into potential therapeutic targets for LIRI. While succinate's accumulation and its effects on mitochondrial function are clearly linked to ROS generation and oxidative stress, it is also possible that succinate plays a complex, context‐dependent role in modulating cell survival pathways, including those associated with apoptosis. The potential role of succinate in I/R injury has been highlighted by several recent studies [19, 21, 22, 23, 24, 25], which emphasise its critical involvement in oxidative stress and mitochondrial damage during this pathological process. These findings are crucial for understanding the mechanisms underlying LIRI and developing strategies to mitigate its damaging effects through targeted therapies aimed at modulating succinate levels and oxidative stress.

## 2 Methods

### 2.1 Human Sample Collection

The Ethical Committee of Shanghai Chest Hospital, Shanghai Jiao Tong University School of Medicine approved all experiments and the use of human peripheral blood samples. Informed consent was obtained in writing from all participants who chose to join the study. The peripheral blood samples were collected from participants via venipuncture during the reperfusion phase, ensuring proper aseptic techniques were followed to prevent contamination. The peripheral serum of patients with LIRI and healthy individuals was collected. Additionally, patients with LIRI were excluded if they had a history of cancer, chronic respiratory diseases, or other conditions that could affect the outcomes of the study. The extent of the patient's condition was evaluated based on established clinical criteria, and patients were classified according to the standard LIRI staging system. The patients included in this study were diagnosed with LIRI due to different clinical conditions, such as pulmonary embolism, organ transplantation and cardiopulmonary bypass. We recognise that each of these conditions may involve distinct injury pathways, which could influence the study outcomes. These potential differences should be taken into account when interpreting the results of our analysis. Table 1 summarises the clinical and demographic characteristics of the study participants. Blood specimens were gathered during the reperfusion phase within 6 h post‐surgery, and control samples were obtained from healthy individuals. The specimens were processed promptly by separating the serum using standard centrifugation methods within 2 h of collection to prevent sample degradation. The serum was subsequently stored at −80°C for future analysis. We have included comprehensive details regarding the specific processing and storage protocols to ensure clarity and reproducibility of the methodology.

**TABLE 1:** Demographic data, comorbidities and laboratory test results of healthy controls and LIRI patients.

Variables	Normal range	Healthy controls (*n* = 60)	LIRI patients (*n* = 60)	*p*
Age (years; mean ± SD)		54.57 ± 12.36	53.57 ± 11.76	0.6615^a^
Male gender (*n*, %)		27	32	0.3612^b^
*Comorbidities (n, %)*				
Sepsis		0	4	0.0419^b^
Hemorrhagic shock		6	4	0.5089^b^
Hypertension		19	22	0.5636^b^
Cerebrovascular disease		8	6	0.5695^b^
*Complete blood count (mean ± SD or median) (interquartile range)*				
White blood cell count (×10^9^/L)	4.0–10.0	7.79 ± 2.53	10.50 ± 2.97	0.0249^a^
Lymphocyte count (×10^9^/L)	0.8–4.0	2.19 ± 0.94	1.51 ± 1.46	0.1251^a^
Neutrophil count (×10^9^/L)	1.8–6.3	5.42 ± 1.52	12.76 ± 5.76	0.0153^a^
Monocyte count (×10^9^/L)	0.12–0.80	0.54 ± 0.38	0.67 ± 0.43	0.4721^a^
Haemoglobin (g/L)	110–170	132.34 ± 38.46	124.36 ± 42.79	0.3076^a^
Platelet count (×10^9^/L)	100–300	186.22 ± 81.34	174.23 ± 94.66	0.7625^a^
*Biochemical test*				
Total plasma protein (g/L)	60–80	68.43 ± 5.11	64.39 ± 6.77	0.2716^a^
Globulin (g/L)	20–30	26.35 ± 5.67	25.71 ± 8.24	0.4937^a^
Albumin (g/L)	40–55	43.25 ± 8.92	41.73 ± 10.81	0.3766^a^
ALT (U/L)	5–40	23.48 ± 10.79	26.35 ± 9.78	0.3974^a^
AST(U/L)	8–40	21.79 ± 9.46	24.83 ± 12.37	0.2488^a^
Blood urine nitrogen (mmol/L)	2.86–7.14	6.71 ± 3.22	6.47 ± 3.94	0.1483^a^
Creatinine (μmol/L)	44–106	72.64 ± 19.64	84.57 ± 22.46	0.0716^a^
Uric acid (μmol/L)	89–428	318.67 ± 114.28	367.49 ± 127.46	0.1164^a^
Lactate dehydrogenase (U/L)	109–243	137.26 ± 47.48	152.37 ± 39.77	0.2947^a^
Brain natriuretic peptide (pg/mL)	< 100	72.46 ± 36.82	95.68 ± 27.62	0.0271^a^
Creatinine kinase (U/L)	26–174	54.76 ± 24.83	62.48 ± 29.46	0.1766^a^
Creatinine kinase‐MB (U/L)	< 18	9.48 ± 6.24	11.42 ± 8.76	0.2796^a^
*Blood gas analysis (mean ± SD)*				
SaO_2_	0.95–0.99	0.97 ± 0.04	0.86 ± 0.34	0.0141^a^
PCO_2_ (mmHg)	35–45	36.76 ± 8.22	54.67 ± 16.33	0.0003^a^
PO_2_ (mmHg)	80–110	11.24 ± 32.34	67.86 ± 28.33	< 0.0001^a^
PH	7.35–7.45	7.36 ± 0.12	7.24 ± 0.29	0.0076^a^
BE (mmol/L)	−2.5 to +3	−0.18 ± 2.07	−5.76 ± 5.84	0.0014^a^
HCO_3_^−^ (mmol/L)	22–28	24.26 ± 5.36	22.71 ± 6.17	0.6748^a^
Lac (mmol/L)	0.5–1.7	0.86 ± 0.55	3.59 ± 1.74	0.0127^a^

*Note:* The data of abnormal distribution are expressed as median and IQR.

Abbreviations: ALT, Alanine aminotransferase; AST, Aspartate aminotransferase.

^a^ Student's *t‐*test.

^b^ χ^2^ test.

### 2.2 Metabolomic Analysis

Metabolomic analysis was conducted to assess the metabolic profile of lung tissues from LIRI mice and serum samples from LIRI patients. To extract metabolites, lung tissue and serum samples were thawed on ice and then homogenised using a mechanical homogeniser. The resulting homogenates were mixed with a solvent system of methanol and chloroform (2:1, v/v), followed by centrifugation to separate the organic and aqueous phases. The organic phase was evaporated under nitrogen gas to concentrate the metabolites, and the extracts were reconstituted in an appropriate solvent for subsequent gas chromatography–mass spectrometry (GC–MS) analysis. The GC–MS system used was a Shimadzu 2010 Plus model (Shimadzu Corporation, Japan), equipped with a capillary column (30 m × 0.25 mm i.d., 0.25 μm film thickness). Chromatography was performed under optimal conditions with an initial column temperature of 60°C, ramped at 10°C/min to 300°C, where it was held for 10 min. The system was calibrated using commercially available standards to ensure accurate quantification. The mass spectra were recorded from m/z 50 to 500, and metabolite identification was performed using standard spectral libraries and custom databases. This approach allowed us to compare the metabolite profiles between the LIRI and control groups with confidence, ensuring reliable and reproducible results. Metabolite clusters were visualised in a heatmap using the heat map package of R software (v.3.6.3).

### 2.3 Animals

Eight‐week‐old male C57BL/6 mice (weight: 20 ± 2 g) were sourced from Charles River (Beijing, China). The animals were housed in an environment with free access to food and water, maintained under a 12‐h light/dark cycle, and kept within a temperature range of 22°C–25°C. All experimental protocols adhered to the standards set by the US National Institutes of Health (NIH) in the Guide for the Care and Use of Laboratory Animals (NIH Publication No. 85–23, revised 1996) and were approved by the Ethics Committee of Shanghai Chest Hospital, Shanghai Jiao Tong University School of Medicine. The mice were euthanized by cervical dislocation under deep anaesthesia, in accordance with ethical guidelines for animal welfare.

### 2.4 Experimental Groups and Treatments

The C57BL/6 mice were allocated randomly into four groups, each consisting of six mice, as follows: (1) Sham Group: Mice underwent thoracotomy, tracheal exposure and immediate chest closure without further intervention. (2) LIRI Group: Mice underwent the lung I/R protocol detailed below. (3) LIRI + IgG Group: Mice were given daily intraperitoneal injections of IgG (1 mg/kg) continuously for 7 days post‐I/R procedure. The surgery followed the same ischemia/reperfusion protocol. (4) LIRI + Ab Group: Mice were administered treatment similar to the LIRI + IgG group, with the difference that they were given a succinic acid neutralising antibody (1 mg/kg, Genscript, Nanjing, China) instead of IgG via intraperitoneal injection. The in vivo hilar clamp model for lung ischemia/reperfusion (I/R), along with anaesthesia and analgesia protocols, was performed according to previously described methods [19]. For anaesthesia, mice received a 50 mg/kg sodium pentobarbital intraperitoneal dose and were kept on a heating pad to maintain a stable body temperature. Before surgical procedures, a subcutaneous injection of buprenorphine (0.1 mg/kg) was administered to minimise pain and stress. Once adequate anaesthesia was confirmed via tracheotomy intubation, the mice were connected to a rodent ventilator (RWD Life Science, Shenzhen, China) set at a 10 μL/g tidal volume and 120 breaths per minute respiratory rate. The I/R group's left hilum was constricted with a non‐traumatic microclamp for 1 h of unilateral ischemia and 2 h of reperfusion after thoracotomy. Administration of IgG or succinate‐neutralising antibodies proceeded during the reperfusion period. Sham animals did not have hilar occlusion, although they had the same anaesthetic and surgery. Following the procedure, the lungs were taken out of the chest cavity. After the right major bronchus had been ligated and 5 mL of sterile, pre‐cooled saline was infused into the left lung, recovery occurred 1 min later. The recovery rate was maintained at a minimum of 70%. Capillary permeability was determined by quantifying the increase in lung weight resulting from higher venous pressure [26, 27]. The perfusion process was repeated three times to get the BALF. The fluid was centrifuged at 3200 × g for 10 min, and the supernatant was collected and stored at −80°C. After processing the left lung, the wet/dry (W/D) ratio was calculated: one section was fixed in 4% paraformaldehyde, another section was weighed, dried for 48 h at 60°C and reweighed. The leftover tissue was then frozen at −80°C for further analysis.

### 2.5 Assessment of Protein Levels in Bronchoalveolar Lavage Fluid

The protein concentration in BALF was measured by using tracheal cannulation to fill the lungs with normal saline. Lavage was performed three times with 0.5 mL sterile saline each, and the recovered fluid was collected and centrifuged at 3000 rpm for 10 min at 4°C to remove cells and debris. The supernatant was carefully collected for protein analysis. According to the provided protocol, protein levels in the BALF were determined using a Bradford Protein Quantification Kit (Beyotime Institute of Biotechnology, China, Cat. No. P0006C). Measurements were conducted following the manufacturer's instructions, and absorbance was read at 595 nm using a microplate reader.

### 2.6 Hypoxia/Reoxygenation (H/R) Treatment and Cell Culture

Human alveolar epithelial cells (Type II AECs) were obtained from Pricella Company (Wuhan, China) and grown in Dulbecco's modified Eagle's medium (DMEM). Enrichment of the culture medium was then carried out with fetal bovine serum (10%, Gibco, Carlsbad, CA, USA) and glutamine (1%). Cells were cultured in an incubator, where hypoxic and reoxygenation conditions were applied at 37°C and 5% CO_2_ (Thermo Fisher Scientific 8000, Marietta, GA). The procedure involved incubating cells for 6 h at 37°C in an environment comprising 5% CO_2_, 1% O_2_ and 94% N_2_. This was followed by reoxygenation for 2 h in a mixture of 95% air and 5% CO_2_, consistent with earlier studies [28]. For succinate or N‐acetyl‐L‐cysteine (NAC) treatment, the cells were incubated with 10 μM succinate (Sigma‐Aldrich) [29] or 0.5 μM NAC (Sigma‐Aldrich) pre‐treatment for 30 min, and then subjected to H/R, as needed.

### 2.7 Mitochondrial Membrane Potential Assessment

The mitochondrial membrane potential (Δψm) was measured using the fluorescent probe JC‐1 (tetrachloro‐tetraethylbenzimidazol carbocyanine iodide) from Invitrogen (USA). After collection and resuspension, Type II AECs were incubated with JC‐1 (200 μM) at 37°C for 20 min. Subsequently, the cells were washed twice with PBS for 30 s each. Data analysis was subsequently performed using a fluorescence microscope (IRX60, Sunnyoptical, Ningbo, China). Fluorescence was detected at 488 nm excitation, with emissions at 530 nm (monomer) and 590 nm (aggregate).

### 2.8 Transmission Electron Microscopy (TEM)

The cells were rinsed briefly with cold PBS, followed by their overnight fixation at 4°C using glutaraldehyde (2.5%) in phosphate buffer (Servicebio, Wuhan, China). After further washes with the buffer, the samples were post‐fixed at room temperature for 2 h with 1% osmium tetroxide in phosphate buffer. Following infiltration, the specimens were embedded in EMbed‐812 resin (Electron Microscopy Sciences) and sectioned. Double staining was performed using lead citrate and uranyl acetate. The specimens were then examined and analysed with a Hitachi H‐7650 transmission electron microscope.

### 2.9 Oxidative Stress Evaluation

To assess ROS levels in Type II AECs, the ROS assay kit (Nanjing Jiancheng Bioengineering Institute, China, Cat. No. E004‐1‐1) was used, following the protocol outlined by Chunli Yang et al. (Cai et al., 2020). A cell suspension was prepared using trypsin digestion, then gently washed twice with PBS to remove residual enzymes and cell debris. To account for potential confounding effects of cell death, cell viability and cell counts were assessed before measuring oxidative stress markers, ensuring that the observed changes were not due to reduced cell numbers. The cells were incubated with 10 μM 2′,7′‐dichlorofluorescein diacetate (DCFH‐DA) at 37°C for 30 min in the dark to allow full uptake of the probe. After incubation, unbound dye was removed by rinsing the cells with serum‐free medium, and ROS levels were measured by flow cytometry according to the kit instructions. Similarly, the levels of catalase (CAT), glutathione peroxidase (GPx), malondialdehyde (MDA) and superoxide dismutase 2 (SOD) were determined using commercial kits purchased from Elabscience (Wuhan, China), including CAT (Cat. No. E‐BC‐K031‐M), GPx (Cat. No. E‐BC‐K096‐M), MDA (Cat. No. E‐BC‐K025‐M) and SOD (Cat. No. E‐BC‐K020‐M). Cells were lysed with the extraction buffer provided in each kit, and the lysates were centrifuged at 3000 rpm for 10 min to collect the supernatants. Subsequent assays were carried out in strict accordance with the manufacturers' protocols, and absorbance values were recorded using a microplate reader.

### 2.10 Assessment of Apoptosis

After treatment, a single‐cell suspension was prepared by incubating the cells with trypsin for 4 min. Following centrifugation, the cells were washed with cold D‐Hanks solution (pH 7.2–7.4) to remove any residual trypsin. The cells were then incubated for 15 min at room temperature with Annexin‐V binding buffer, containing Annexin‐V‐FITC and propidium iodide (PI), which allowed identification of both early and late apoptotic cells. Fluorescence from Annexin‐V‐FITC and PI was detected using a flow cytometer (Becton Dickinson, United States). The settings included 488 nm excitation and 530 nm emission for Annexin‐V‐FITC, and 488 nm excitation and 617 nm emission for PI. At least 10,000 cells per group were analysed. Cell populations were gated to eliminate debris and doublets, and apoptotic cells were categorised based on Annexin‐V and PI staining. Early apoptotic cells were defined as Annexin‐V positive and PI negative, while late apoptotic or necrotic cells were both Annexin‐V and PI positive.

### 2.11 Oxygen Consumption Rate Measurement

The Seahorse XF24 analyser (Seahorse Bioscience Inc., Boston, Massachusetts, USA) determined the oxygen consumption rate (OCR) in Type II AEC cells. After inoculating XF24‐well cell culture plates with Type II AECs and letting the Seahorse XF basal medium equilibrate, the OCR was measured. Oligomycin (10 μM), FCCP (1 μM), rotenone (0.5 μM) and antimycin A (0.5 μM) were used during the assay to inhibit specific mitochondrial complexes as per standard protocols. The OCR data were measured in pmol/min (picomoles per minute) and normalised to the total protein content in each sample to account for any variations in cell number or protein expression. This normalisation step was crucial for ensuring accurate comparisons of mitochondrial function across the experimental conditions. The OCR was established following the manufacturer's guidelines.

### 2.12 Western Blotting

Protein extraction was performed using RIPA buffer (Cell Signalling Technology, Cat. No. 9806) supplemented with protease and phosphatase inhibitors (Thermo Fisher, Cat. No. 78442). The protein concentration was determined using the Bradford Protein Quantification Kit (Beyotime Institute of Biotechnology, Cat. No. P0006C), according to the manufacturer's instructions. Proteins were separated by SDS‐PAGE using 10% polyacrylamide gels. After separating and electrotransferring 30 μg of protein from each cell and tissue sample onto polyvinylidene difluoride membranes, primary antibodies were used to probe the membranes, including acyl‐CoA oxidase 1 (ACOX1; Proteintech, Cat No. 83731‐2‐RR, 1:30000), isocitrate dehydrogenase 2 (IDH2; Proteintech, Cat No. 15932‐1‐AP, 1:2000), β‐actin (Proteintech, Cat No. 66009‐1‐Ig, 1:50000). The membranes were incubated overnight at 4°C with primary antibodies, diluted in 5% nonfat milk. After incubation, the membranes were washed three times with Tris‐buffered saline with 0.1% Tween‐20 (TBS‐T) for 15 min each. The membranes were then treated with HRP‐conjugated secondary antibodies (anti‐mouse/rabbit antibodies, Cell Signalling Technology, dilution 1:5000). Following three additional washes with TBS‐T (20 min per wash), the immunological complexes were visualised using enhanced chemiluminescence. ImageJ software (NIH, Bethesda, MD) was applied to quantify and analyse the band intensities.

### 2.13 Haematoxylin and Eosin (H&E) Staining

Lung tissues were harvested and fixed in 4% paraformaldehyde for 24 h at ambient temperature. Following fixation, the tissues were embedded in paraffin and sectioned into 3 μm slices using a microtome. The sections were deparaffinised using xylene and rehydrated through a series of graded ethanol solutions. Afterward, the sections were stained with haematoxylin for 5 min, followed by eosin for 2 min. The tissue was then dehydrated and mounted for further examination. Lung injury was evaluated by examining histopathological alterations in lung tissue, with a scoring system based on four key criteria: alveolar edema, haemorrhage, inflammatory cell infiltration and alveolar wall thickening. Each criterion was scored on a scale from 0 to 4, where 0 indicated no injury and 4 represented the most severe damage. The scores for each criterion were summed to provide an overall lung injury score, allowing for a comprehensive assessment of the extent of damage caused by I/R.

### 2.14 TUNEL Assay

Consistent with the previously described method, the TUNEL assay was performed using a one‐step TUNEL reagent (C1089, Beyotime Institute) [30]. In brief, each group's lung tissue section (3 μm) was dewaxed. Following a 30‐min incubation at 37°C, the section was maintained with 20 μg/mL of protease K (without DNase). Following PBS washing, tissue slices were exposed to a TUNEL reaction mixture (50 μL) and incubated in the dark for 60 min at 37°C. After staining, the sections were examined under a fluorescence confocal microscope (Zeiss LSM710, Germany). Fluorescence was observed with excitation at 488 nm and emission at 530 nm. The TUNEL‐positive cells in 10 randomly selected fields were quantified using ImageJ software (Bio‐Rad Laboratories, Hercules, CA, USA).

### 2.15 Statistical Analysis

The data were analysed with SPSS version 20.0 (IBM Corp., Armonk, NY, USA) and expressed as means ± standard error of the mean from at least three independent trials, each performed in triplicate (or as specified). Two‐tailed Student's t‐test was employed for comparing the means of the two groups. ANOVA was applied for comparisons across three or more groups, followed by Bonferroni's multiple comparisons. A *p*‐value of less than 0.05 was considered statistically significant.

## 3 Results

### 3.1 Succinate Was Increased in LIRI

**FIGURE 1:**
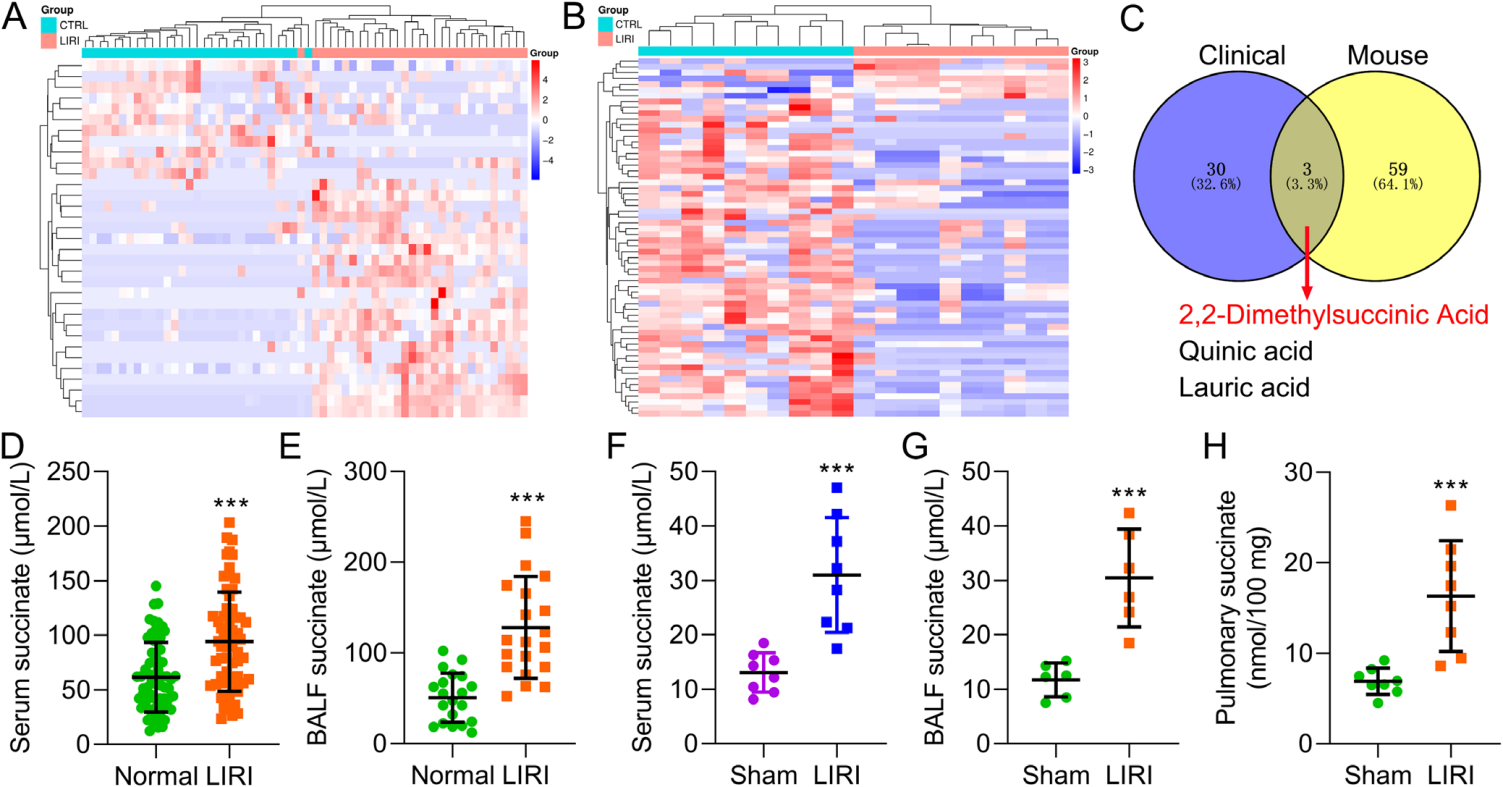
Succinate was increased in LIRI. (A, B) Heat maps showing the log_2_‐transformed abundances of metabolites selected by metabolomics analysis in peripheral serum of LIRI patients vs. healthy participants (A) and lung tissue of LIRI mice vs. control (B). (C) Venn diagram showing the overlap of metabolites identified in the clinical samples of LIRI patients and in the mouse LIRI model. Three metabolites were identified in both groups, including 2,2‐dimethylsuccinic acid, quinic acid and lauric acid. (D, E) The level of succinate in the LIRI patients' serum and bronchoalveolar lavage fluid (BALF) was measured, with the control group (healthy individuals) being used for normalisation. (F‐H) The expression levels of succinate in the LIRI mice's serum, BALF and lung tissue were measured using the standardised ELISA kits. Data are presented as the means ± standard error of the mean for three independent experiments. ***p < 0.001.

After establishing a mouse model of LIRI, we assessed changes in pulmonary histopathology. Lung I/R damage caused considerable inflammatory cell infiltration, interstitial edema, pulmonary capillary congestion and thickening of the alveolar wall, as seen by H&E staining (Figure S1A). The filtration coefficient (Kf), protein concentration in bronchoalveolar lavage fluid (BALF) and the lung wet‐to‐dry (W/D) weight ratio—important markers of pulmonary vascular permeability and acute lung injury—were evaluated. The group subjected to lung ischemia/reperfusion (I/R) showed an increase in the W/D ratio, Kf and BALF protein concentration compared to the control group (Figure S1B–D). The quantitative histological injury score was increased, and the injury was characterised by severe edema, haemorrhage and inflammatory infiltration, consistent with the scoring system described earlier (Figure S1E). These results validate the LIRI mouse model's successful establishment. Then, using metabolomics techniques, we examined the expression of differential metabolites in the lung tissues of LIRI mice and the serum of LIRI patients. This analysis identified a significant increase in metabolites associated with LIRI. In LIRI patients, the top five increased metabolites were glycolic acid, Analyte 497, 1,5‐Anhydroglucitol, Mono(2‐ethylhexyl)phthalate and arbutin. Conversely, the top five decreased metabolites included citraconic acid, uracil, carbazole, Methyl Phosphate and 2‐amino‐3‐(4‐hydroxyphenyl)propanoic acid; In LIRI mice, the most increased metabolites were guanosine‐5′‐monophosphate, thymidine, Hesperitin, 4‐hydroxycinnamic acid and 2‐ketoadipate. The top five decreased metabolites included benzoylformic acid, 2‐keto‐isovaleric acid, quinic acid, aniline and lactose (Figure 1A,B; Tables S1 and S2). Recent studies have identified succinate accumulation as a pathogenic hallmark and important metabolic signature linked to increased generation of ROS during I/R injury [19, 20]. Figure 1C shows a Venn diagram illustrating the overlap of metabolites identified in both clinical LIRI patient samples and the LIRI mouse model. The diagram highlights three key metabolites, 2,2‐dimethylsuccinic acid, quinic acid and lauric acid, which were commonly elevated in both groups, suggesting a shared metabolic pathway in the development of LIRI. The limited overlap of metabolites could be attributed to differences in metabolic processes between species, as well as the diverse clinical characteristics of the patient cohort. Factors such as underlying health conditions, medications, and the stage of the disease may all contribute to variations in the metabolic profiles observed. To confirm this, we analysed succinate levels in the serum and BALF of patients with LIRI. The findings showed a significant increase in succinate concentrations in peripheral serum and BALF (Figure 1D,E). Likewise, succinate levels were measured in LIRI mice's lung, BALF and serum, and consistent findings were observed (Figure 1F–H). These results suggest that succinate is increased in LIRI and might be important to the disease's development.

### 3.2 Succinate Promotes H/R‐Induced Pulmonary Cell Apoptosis and Oxidative Stress

**FIGURE 2:**
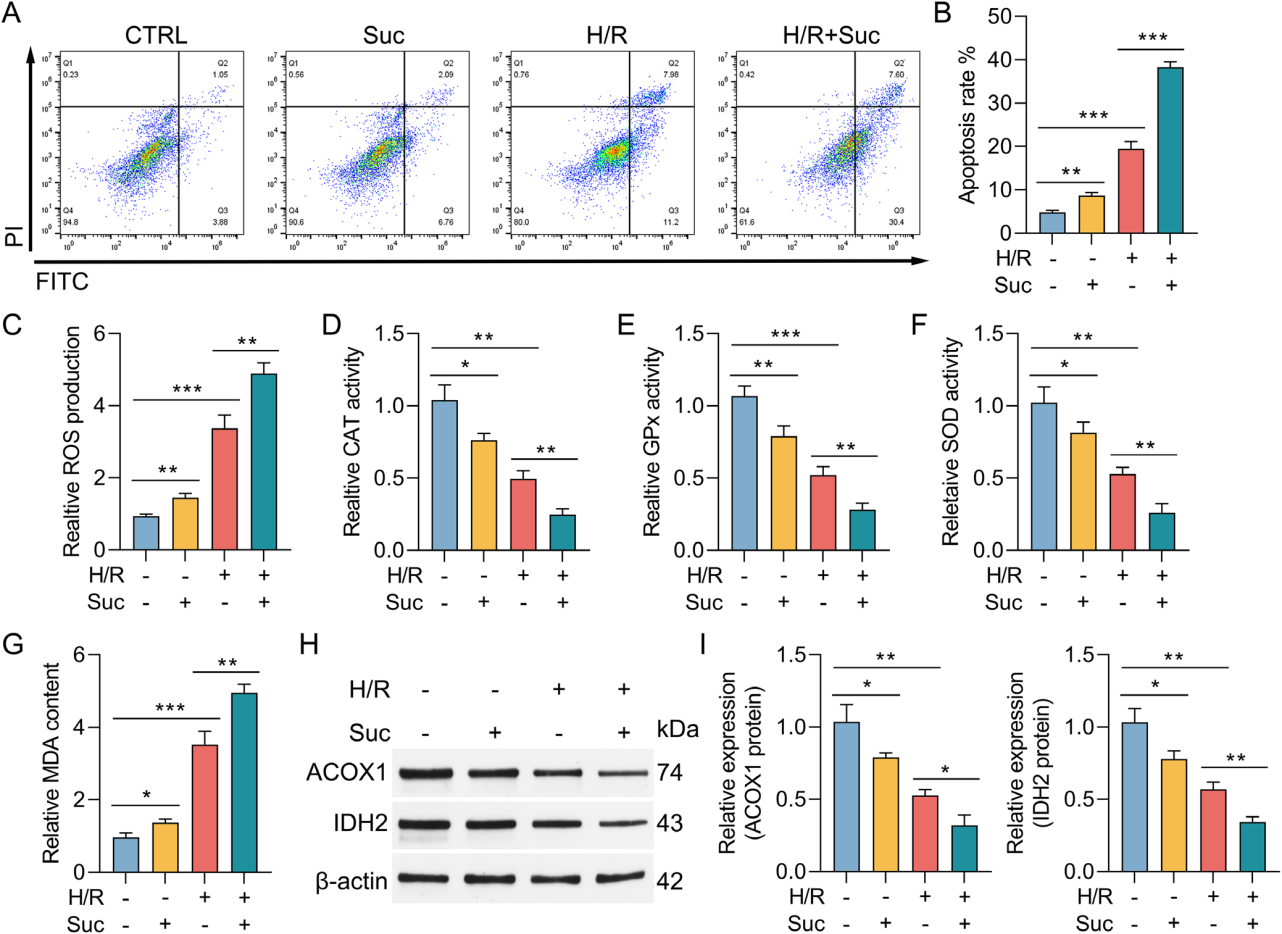
Succinate promotes H/R‐induced pulmonary cell apoptosis and oxidative stress. (A) Representative FACS dot plots showing annexin V and propidium iodide dual staining. (B) FACS analysis of apoptosis rates in Type II AECs. (C–G) Measurement of ROS, CAT, GPx, SOD and MDA concentrations in Type II AECs from each group. (H) Each group has representative bands of ACOX1 and IDH2 of Type II AECs. (I) The relative band intensity of ACOX1 and IDH2 in each group. Band intensity was quantified using ImageJ software and normalised to the control group. Data are presented as the means ± standard error of the mean for three independent experiments. *p < 0.05, **p < 0.01 and ***p < 0.001.

We established a commonly used H/R model that simulates lung ischemia and blood restoration to determine the molecular and cytopathological changes induced by I/R using previously reported methods [28]. As shown in Figure 2A,B, H/R treatment led to an increase in cell apoptosis compared with the control group, while the apoptosis rates detected using FACS were increased in the H/R + succinate group compared to those of the H/R group. To rule out the possibility that the observed changes in oxidative stress markers and apoptosis rates were simply due to a reduction in cell numbers, we assessed cell viability and performed cell counts before measuring these parameters. Then, the degree of oxidative stress was evaluated by measuring the levels of ROS, CAT, GPx, SOD and MDA in Type II AECs. As shown in Figure 2C–G, after suffering H/R, the levels of ROS and MDA in Type II AECs were increased, while the levels of SOD, CAT and GPx were decreased, suggesting that the H/R process caused violent oxidative stress in Type II AECs cells. Importantly, we found that succinate could accelerate oxidative stress induced by H/R. Additionally, western blotting was conducted to assess the expression of ACOX1 and IDH2 in Type II AECs. These enzymes play a crucial role in regulating mitochondrial metabolism and oxidative stress [31, 32], making them key targets for investigating the effects of succinate on mitochondrial dysfunction in the context of LIRI. H/R injury led to a reduction in the protein expression levels of ACOX1 and IDH2. Notably, succinate pre‐treatment further decreased the expression levels of ACOX1 and IDH2 (Figure 2H,I). These findings suggest that succinate exacerbates H/R‐induced pulmonary cell apoptosis and oxidative stress.

### 3.3 Succinate Impairs Mitochondrial Function in Alveolar Epithelial Cells After H/R

**FIGURE 3:**
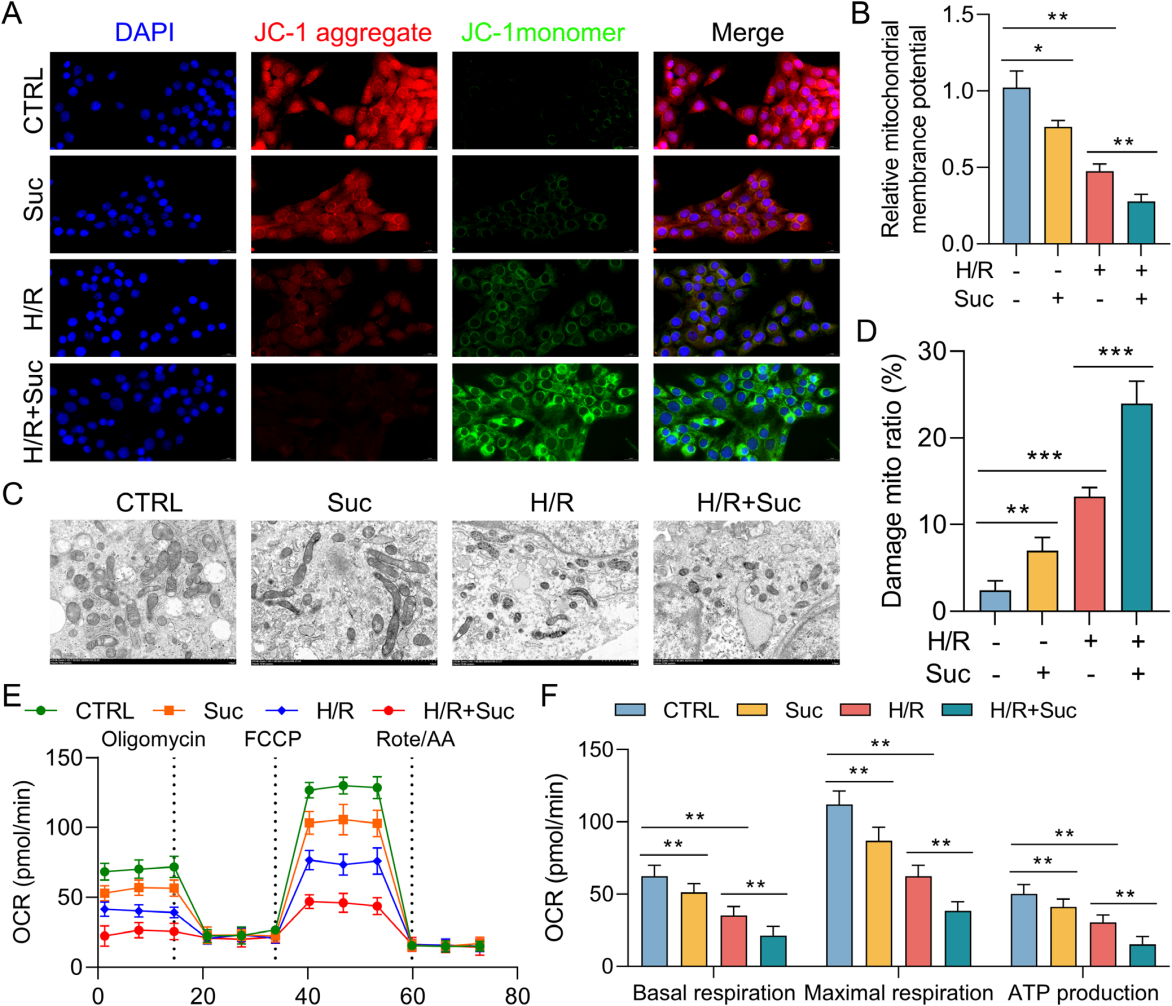
Succinate impairs mitochondrial function in alveolar epithelial cells after H/R. (A) Representative images showing MMP (fluorescent staining) in Type II AECs for each group using JC‐1 staining. (B) A histogram illustrating MMP depolarization determined by the red/green fluorescence ratio. (C) TEM images of mitochondria after H/R injury in Type II AECs (magnification 2000×). (D) Quantification of mitochondrial damage in each group, evaluated by examining mitochondrial morphology and identifying specific markers of damage, including swelling, membrane rupture and loss of cristae. (E) Mitochondrial OCR in Type II AECs across groups (with oligomycin at 10 μM, FCCP at 1 μM and rotenone/antimycin A at 0.5 μM) was measured and reported in pmol/min, using the Seahorse XF Analyser. (F) Basal respiration, maximal respiration and ATP production in Type II AECs for each group. Data are expressed as means ± standard error of the mean from three independent experiments. *p < 0.05, **p < 0.01, ***p < 0.001.

Although IR‐induced lung damage has been associated with mitochondrial dysfunction in several previous studies, we postulated that succinate increases this dysfunction. The reduction of mitochondrial membrane potential (Δψm) and the activation of the mitochondrial permeability transition pore (mPTP) are prominent markers of mitochondrial dysfunction. The JC‐1 fluorescent dye assay showed that the H/R insult substantially reduced mitochondrial membrane potential compared to the control group, as shown in Figure 3A,B. In addition, the succinate‐treated group had a substantially reduced mitochondrial membrane potential compared to the H/R group. To assess the influence of succinate on mitochondrial morphology, the H/R injury‐exposed AECs were analysed using TEM. Cells treated with H/R showed fragmentation, cristae loss and mitochondrial distension in TEM images. In contrast to the H/R group, these morphological changes were exacerbated in the succinate‐treated group (Figure 3C,D). The mitochondrial OCR was assessed as well to evaluate functional damage. Compared to the H/R group, the succinate‐pretreated Type II AECs had a considerably reduced mitochondrial OCR (Figure 3E,F). The data demonstrate that succinate increases H/R‐induced mitochondrial dysfunction in Type II AECs.

### 3.4 NAC Retarded Succinate–Induced Cell Apoptosis and ROS

**FIGURE 4:**
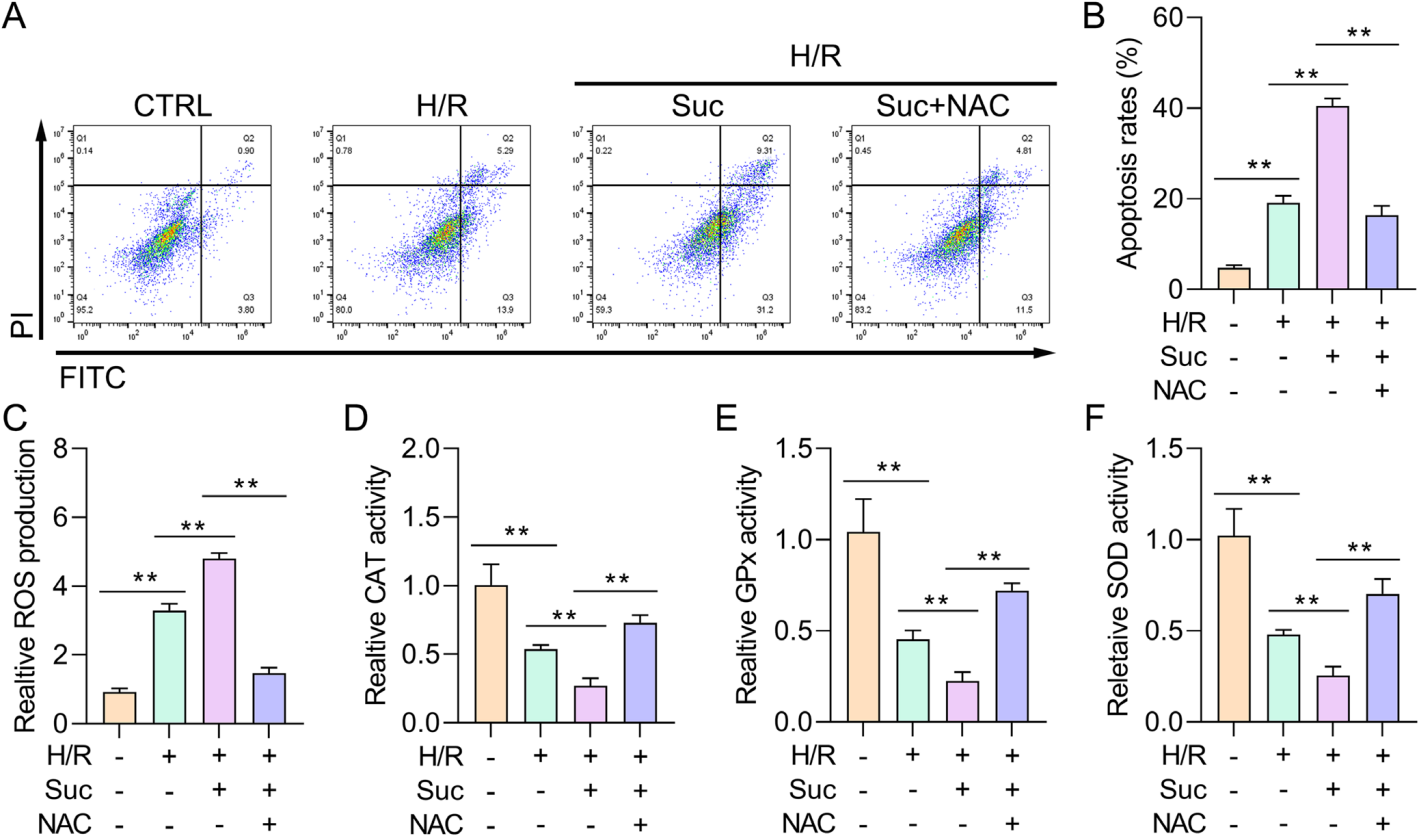
NAC retarded succinate‐induced cell apoptosis and ROS. (A, B) Type II AECs apoptosis was determined by FCM assay in different groups (*n* = 3). (C–F) The activities of different oxidases in different treated Type II AECs were detected. **p < 0.01.

The ability of NAC to suppress ROS is well recognised. To assess whether the ROS scavenger NAC may reduce succinate‐induced apoptosis in Type II AECs, before succinate exposure, cells were pretreated with 0.5 μM NAC for 30 min. Flow cytometry analysis showed succinate treatment substantially increased apoptosis in Type II AECs. However, NAC pretreatment reduced this apoptotic response (Figure 4A,B). To further determine oxidative stress across the various experimental groups, levels of ROS and the levels of the key antioxidant enzymes—SOD, CAT and GPx were—evaluated. The results indicated that succinate exposure increased intracellular ROS levels and decreased the activities of SOD, CAT and GPx. By lowering ROS levels and restoring antioxidant enzyme activity, NAC treatment, on the other hand, reduced these changes (Figure 4C–F). The results indicate that succinate induces cell apoptosis and ROS formation in Type II AECs, but NAC effectively reduces these effects by decreasing ROS while sustaining antioxidant defence mechanisms.

### 3.5 NAC Improved Succinate‐Induced Mitochondrial Dysfunction in Type II AECs After H/R

**FIGURE 5:**
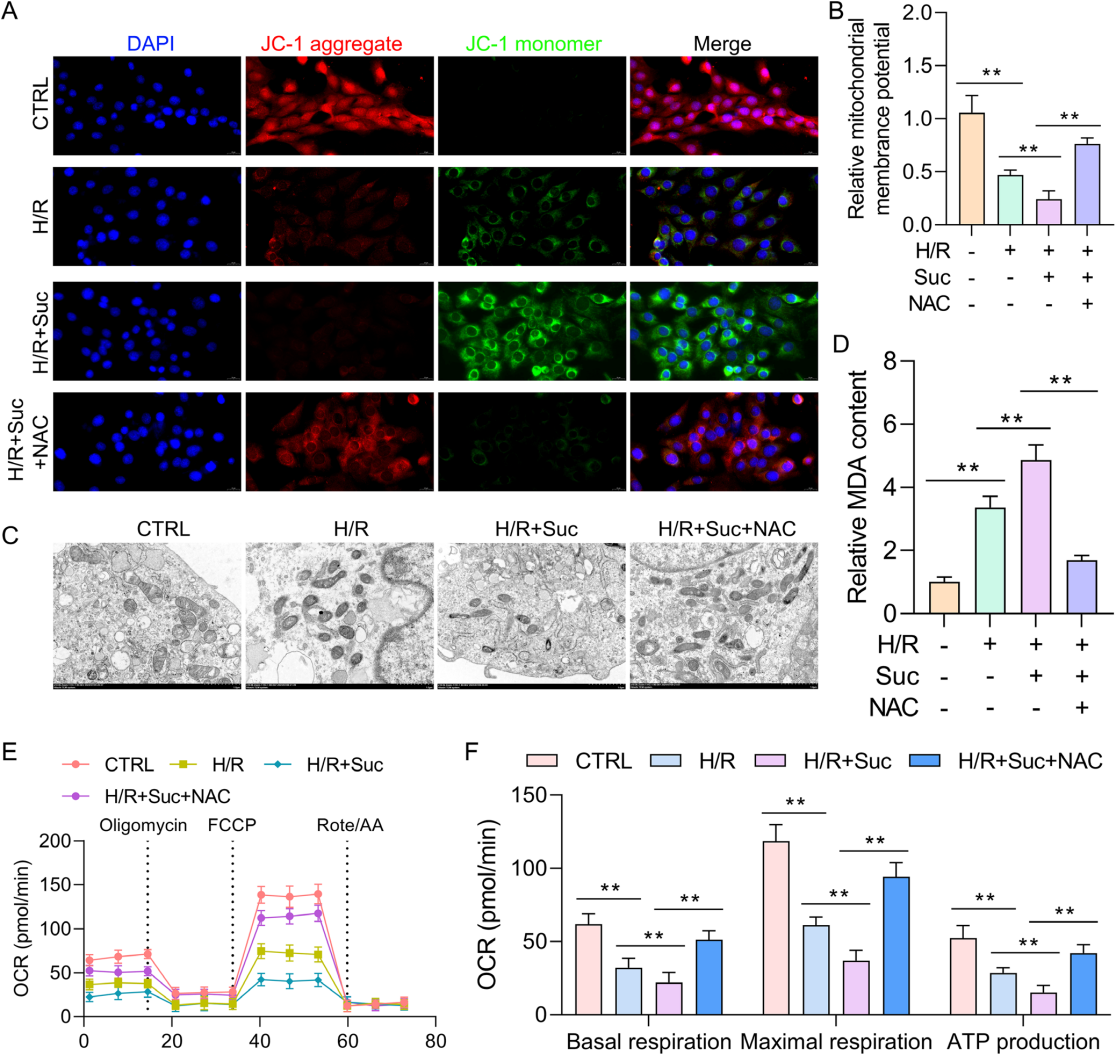
NAC improved succinate‐induced mitochondrial dysfunction in Type II AECs after H/R. (A) Representative images showing fluorescent staining of Type II AECs in all groups. (B) Mitochondrial membrane potential in Type II AECs for each group, with a histogram depicting the red/green fluorescence ratio to measure membrane depolarisation. (C) TEM images of mitochondria from H/R‐treated Type II AECs in each group (magnification 2000×). (D) Quantification of mitochondrial damage in each group. (E) OCR in Type II AECs across all groups was quantified and expressed in pmol/min, measured using the Seahorse XF Analyser. (F) Basal respiration, maximal respiration and ATP production levels in Type II AECs across the groups were assessed using the Seahorse XF Analyser. Data are expressed as means ± standard error of the mean from three independent experiments. **p < 0.01.

The MitoTracker was utilised for immunofluorescence targeting mitochondria in Type II AECs exposed to H/R to evaluate mitochondrial morphology further. The findings showed that H/R + Suc decreased the mitochondrial membrane potential compared to the H/R group. Instead of H/R + Suc, H/R + Suc + NAC had a greater mitochondrial membrane potential (Figure 5A,B). The H/R + Suc mitochondria were consistently rounder, smaller and fragmented, as determined by TEM. Conversely, the H/R + Suc + NAC group's mitochondria were longer and more interconnected (Figure 5C). The MDA, a lipid peroxidation marker, was used to determine the degree of oxidative stress and validate these results. Following H/R treatment, Type II AECs had considerably higher MDA levels than the control group, as shown in Figure 5D. Treatment with succinate further increased MDA levels, suggesting that succinate exacerbates oxidative stress by H/R. However, the oxidative stress induced by succinate was considerably reduced by NAC co‐treatment. Seahorse analysis was also used to analyse OCR to measure mitochondrial function. The H/R + Suc group showed a substantial decrease in maximum respiration, ATP generation and basal OCR. Conversely, co‐treatment with NAC (H/R + Suc + NAC) enhanced OCR and ATP synthesis in Type II AECs (Figure 5E,F). Lastly, ROS‐regulating protein expression was investigated while employing the western blot. The H/R + Suc group had considerably greater IDH2 and ACOX1 concentrations than the H/R group. The significant decreases in IDH2 and ACOX1 induced by succinate were partially reversed by NAC treatment (Figure S2). Thus, results suggest NAC reduces the mitochondrial dysfunction caused by succinate in Type II AECs following H/R.

### 3.6 Succinate Aggravates the I/R‐Induced Lung Injury and Inflammation In Vivo

**FIGURE 6:**
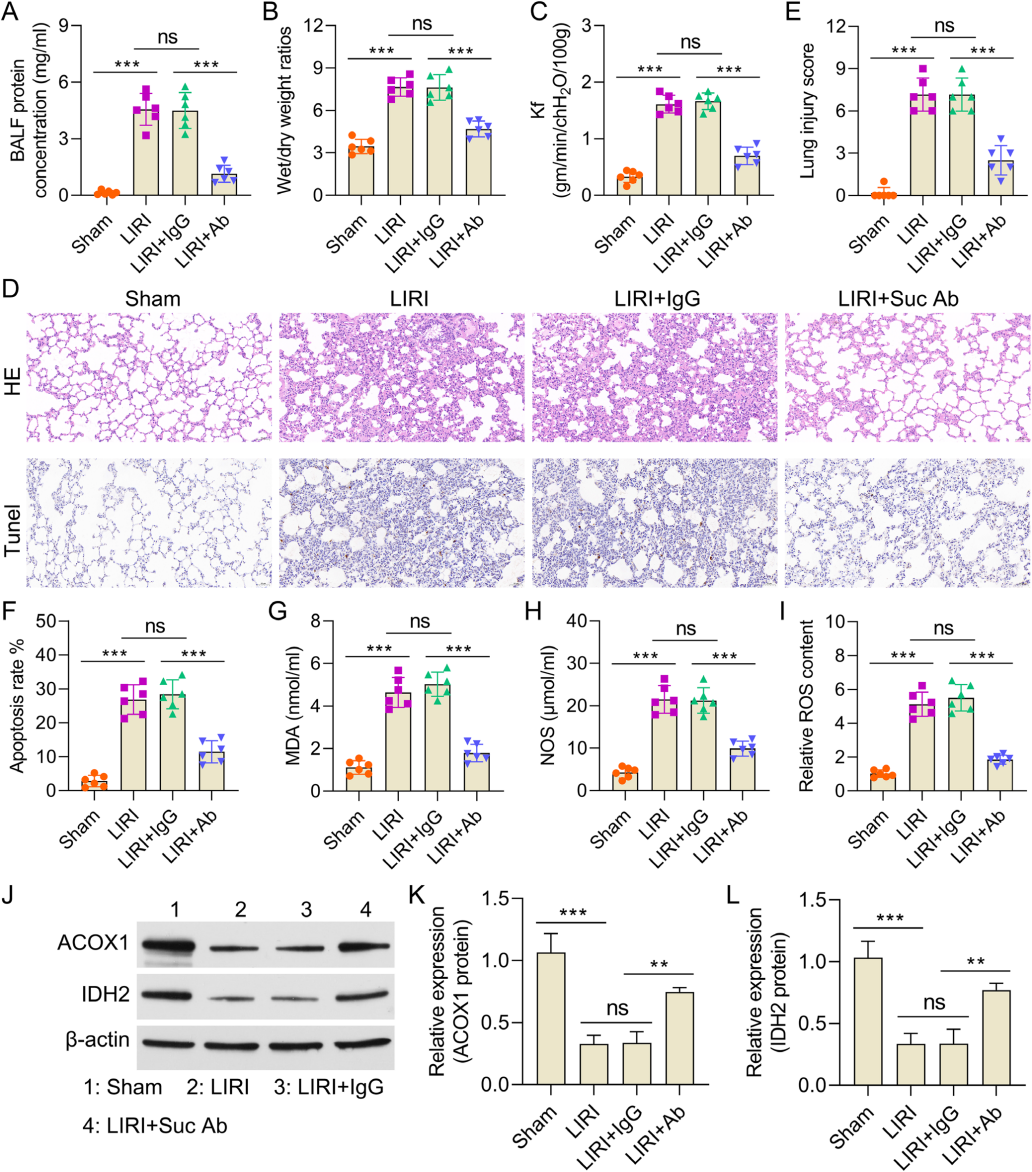
Succinate aggravates the I/R‐induced lung injury and inflammation in vivo. (A) Levels of BALF protein in lung tissues. (B) Wet‐to‐dry ratios (C) capillary permeability in different groups. (D) Representative lung tissue sections stained by H&E and TUNEL. (E) Lung injury scores. (F) The apoptosis rates and (G–I) the activities of different oxidases in each group of mice were detected. (J) The expression of IDH2 and ACOX1 in each group. (K, L) The relative band intensity of ACOX1 and IDH2 in each group. Data are presented as the means ± standard error of the mean for three independent experiments. **p < 0.01 and ***p < 0.001.

A mice lung I/R model was established using C57BL/6 mice to analyse the function of succinate in LIRI. The LIRI + IgG group had higher protein contents in BALF than the sham group, which was reversed by treatment with a succinate antibody (LIRI + succinate Ab) (Figure 6A). The lung wet‐to‐dry weight ratio was used to measure perivascular edema, and it was greater in the LIRI + IgG group. Treatment with succinate antibodies reduced this ratio, suggesting that inhibiting succinate reduces I/R‐induced pulmonary edema (Figure 6B). Capillary permeability measurements showed similar results (Figure 6C). Normal histology was seen in sham‐operated mice lung tissue. Conversely, I/R‐induced injury causes intra‐alveolar bleeding, severe interstitial edema and high neutrophil infiltration, which was seen in the LIRI + IgG group. H&E staining demonstrated that succinate antibody therapy reduced these clinical changes (Figure 6D). Furthermore, the succinate antibody mitigated the increased lung damage scores associated with I/R (Figure 6E). Comparing the lung tissues of the LIRI + IgG group to the sham group, TUNEL testing showed increased apoptosis. The increase was substantially lowered by succinate antibody treatment (Figure 6D,F). Moreover, the concentrations of oxidative stress indicators, such as MDA, NOS and ROS, were greatly increased in the LIRI + IgG group compared to the sham group. The succinate antibody treatment decreased these levels (Figure 6G–I). The expression of ACOX1 and IDH2 was substantially decreased by I/R, as determined by Western blot analysis of lung tissue; both were restored by succinate antibody treatment (Figure 6J–L). These findings collectively indicate that succinate exacerbates I/R‐induced lung damage and inflammation in vivo, while the inhibition of succinate efficiently reduces these pathological effects.

## 4 Discussion

This study highlights the key role of succinate in LIRI. Succinate accumulation contributes to oxidative stress, mitochondrial dysfunction and apoptosis in lung cells. Treatment with NAC reduced succinate‐induced damage, while succinate‐neutralising antibodies mitigated lung injury and inflammation in vivo. These findings suggest that targeting succinate accumulation could be a promising therapeutic approach to reduce oxidative damage and improve lung function after ischemia–reperfusion.

Oxidative stress is a key factor in the development of LIRI, where ischaemic conditions cause a loss of energy and the buildup of harmful oxygen metabolites [33]. Upon reperfusion, these metabolites generate an overproduction of ROS, which in turn leads to mitochondrial dysfunction, including a decline in ATP production and disruption of mitochondrial activity, worsening pulmonary damage [10, 11, 34]. Moreover, apoptosis, driven by ROS and oxidative stress, is intricately linked to LIRI, with mitochondria playing a pivotal role in initiating this process [35]. The accumulation of ROS further destabilises mitochondrial function, elevates intracellular calcium, impairs mitochondrial membrane potential and activates caspases, resulting in intensified tissue injury [36]. As such, addressing mitochondrial dysfunction and oxidative stress could offer effective therapeutic strategies for LIRI.

Metabolomics serves as a valuable approach for investigating metabolic alterations related to diseases like ischemia–reperfusion injury (I/R). In this study, we applied metabolomics to pinpoint crucial metabolic markers in LIRI, focusing on metabolites such as 2,2‐dimethylsuccinic acid, quinic acid and lauric acid. The choice of these metabolites was driven by their significant elevation in both LIRI patient samples and mouse models, along with their involvement in oxidative stress and mitochondrial dysfunction observed during LIRI. Succinate, in particular, emerged as a notable metabolic shift, aligning with previous findings and being associated with mitochondrial damage and increased oxidative stress in the context of LIRI. Our results indicate that succinate facilitates the production of reactive oxygen species (ROS), aggravates mitochondrial injury and amplifies oxidative stress, making it a key factor in the development and progression of LIRI. The rise in quinic acid and lauric acid may contribute to the oxidative stress and inflammatory pathways in LIRI, with quinic acid playing a role in amino acid metabolism and oxidative stress modulation, while lauric acid is involved in immune responses and lipid metabolism. These metabolites could significantly influence immune regulation and inflammation during LIRI. Our analysis also revealed notable metabolic differences between LIRI patients and mouse models, with species‐specific variations in the metabolites detected. For example, 1,5‐anhydroglucitol and Mono(2‐ethylhexyl) phthalate were more prevalent in LIRI patients, while thymidine and hesperidin were elevated in the mouse model. These discrepancies may arise from inherent differences in metabolic processes between species or be influenced by clinical factors such as health status, medication use and disease progression. Additionally, the limited overlap of metabolites between humans and mice underscores the need to carefully interpret data when attempting to extrapolate findings from animal models to human conditions.

Succinyl‐CoA is converted to succinic acid, a TCA cycle metabolite, which is then oxidised to fumarate [37]. Succinate levels, often confined to the mitochondrial matrix, are strictly controlled. There is growing evidence that the buildup of succinate is a pathogenic characteristic and an important metabolic signature of excessive ROS generation during I/R injury [38]. The decrease of the coenzyme Q (CoQ) reserve during ischemia inhibits succinate oxidation, causing succinate to accumulate. Furthermore, reversing succinate dehydrogenase (SDH) activity under ischemic circumstances promotes the conversion of fumarate to succinate [39]. The inhibition of SDH activity during ischemia results in succinate accumulation, and its subsequent oxidation upon reperfusion further exacerbates ROS production [19].

The oxidation of stored succinate by SDH after reperfusion induces a mitochondrial ROS burst, exacerbating mitochondrial injury. In our study, we observed a significant upregulation of succinate in LIRI, which further confirmed its role in ROS generation. We also noted that this accumulation of succinate is closely linked to the oxidative damage and mitochondrial dysfunction observed in lung cells. While our study suggests that succinate accumulation plays a critical role in ROS generation, we did not specifically investigate its exact localization within the mitochondrial matrix. We recognise that succinate, as an electron donor at complex II, likely contributes to ROS production during LIRI by accumulating in the matrix. Reverse electron transfer (RET) at mitochondrial complex I, triggered by succinate oxidation, has been widely linked to excessive ROS generation. As demonstrated, RET amplifies ROS production compared to forward electron transport [40, 41]. This mechanism further supports the role of succinate in driving ROS production during reperfusion. Future studies should focus on identifying the precise sites of succinate accumulation and their direct association with ROS generation at complex II.

The precise effect of succinate on LIRI is undetermined, even though its participation in I/R injury has been shown in the mouse kidney [25]. This research found an increase in ROS and MDA levels, while the activities of antioxidant enzymes, such as SOD, CAT and GPx, decreased in type II AECs after H/R. These results demonstrate that hypoxia/reoxygenation produces significant oxidative damage in alveolar epithelial cells. Succinate was reported to accelerate oxidative stress and apoptosis via a mitochondria‐dependent mechanism. Treatment with NAC, a ROS scavenger, successfully inhibited succinate‐induced dysfunction of the mitochondria, decreasing ROS generation. NAC, known for its role as a ROS scavenger, also enhances glutathione synthesis, which contributes to the activation of antioxidant enzymes, including CAT, SOD and GPx. This dual effect may explain the increase in these enzymes' activity observed after NAC treatment. The infusion of a succinate‐neutralising antibody in vivo reduced oxidative stress in the lungs of mice exposed to H/R. The findings indicate that NAC and succinate‐neutralising antibodies may reduce succinate‐induced ROS overproduction, reduce oxidative stress and protect lung cells against H/R‐induced injury.

Succinate's accumulation during ischemia and its subsequent oxidation during reperfusion is a crucial driver of ROS generation, as it triggers SDH activation, resulting in a burst of ROS that exacerbates mitochondrial dysfunction [19]. This phenomenon, although central to our study, has been well‐documented in various tissues, suggesting that succinate accumulation is a broader characteristic of IR injury. For instance, research by Folbergrova et al. [42], Sahni et al. [43], Nishima and Tanaka [44] and Schlegel et al. [19] has shown that succinate accumulates in ischemic brain, kidney and liver tissues, contributing to mitochondrial dysfunction and increased ROS production. These studies emphasise the relevance of succinate as a metabolic driver of oxidative stress during IR injury. Our study extends these findings by exploring the specific role of succinate in LIRI. By highlighting succinate's involvement in oxidative stress and mitochondrial damage in the lungs, we provide a deeper understanding of its metabolic effects and the potential for therapeutic interventions targeting succinate accumulation in future studies.

While previous studies have indicated that succinate plays a role in mitochondrial dysfunction during ischemia/reperfusion in various organs such as the heart and kidney [19, 24, 25], our study is the first to comprehensively explore the specific mechanisms of succinate in LIRI, utilising both clinical samples and in vivo models. We have clarified how succinate enhances oxidative stress and apoptosis via mitochondrial‐dependent pathways. In addition, we have shown that antioxidant therapies, including NAC and succinate‐neutralising antibodies, can reduce lung injury caused by H/R. These results provide new therapeutic perspectives for targeting succinate‐induced ROS overproduction as a strategy to mitigate LIRI. Although succinate's effects have been explored in other organs, this study deepens our understanding of its distinct role in the lung and presents new opportunities for therapeutic intervention in LIRI.

The current study has several limitations. While our study confirms the protective role of succinate‐neutralising antibodies, it does not delve into the specific mechanisms by which these antibodies reach the mitochondrial matrix and interact with succinate. Although I/R may lead to alterations in mitochondrial membrane permeability that could facilitate antibody entry, the precise intracellular transport processes remain undefined. Moreover, we acknowledge that a significant limitation of this study is the lack of direct evidence showing that the succinate‐neutralising antibody reduces succinate accumulation. While the antibody improves various injury hallmarks of LIRI, the exact mechanism remains uncertain. An additional limitation of our study is the systemic administration of the succinate‐neutralising antibody. While no significant undesirable effects were observed in other tissues, we acknowledge that the possibility of off‐target effects exists. Future studies should investigate the potential impact of succinate‐neutralising antibodies on other organs and tissues to assess any unintended consequences. Further studies are needed to clarify the pathways responsible for antibody localisation within mitochondria and to elucidate their exact function in counteracting succinate‐induced oxidative stress. Another limitation of this study is the use of GC–MS for metabolic analysis, particularly in measuring 2,2‐dimethylsuccinic acid, which is not a direct precursor of succinate. The method was not fully detailed, potentially affecting the accuracy and reproducibility of the results. Future studies should provide a more comprehensive description of the methodology. A key issue is the lack of in‐depth analysis of the metabolic pathways associated with the increased metabolites in LIRI. Future research should explore these pathways to better understand the roles these metabolites play in LIRI progression. Additionally, the use of the JC‐1 staining method to assess mitochondrial membrane potential presents limitations, as it may not be entirely reliable due to various influencing factors. Future studies should incorporate alternative methods to validate these findings more effectively. Furthermore, although NAC reduced apoptosis and oxidative stress in Type II AECs, its specificity in mitigating succinate‐induced damage remains unclear. Future research using a succinate dehydrogenase inhibitor like dimethyl malonate would help isolate succinate's role and clarify the underlying mechanisms.

## Author Contributions

**Wenhao Wang:** data curation (equal), formal analysis (equal), validation (equal), writing – original draft (lead), writing – review and editing (equal). **Nana Feng:** investigation (lead), visualization (equal), writing – original draft (equal), writing – review and editing (equal). **Qi Shi:** investigation (equal), writing – review and editing (equal). **Jichun Yang:** data curation (equal), funding acquisition (lead), writing – review and editing (equal). **Yulong Tan:** formal analysis (equal), investigation (equal), writing – review and editing (equal). **Wenyong Zhou:** funding acquisition (equal), methodology (equal), project administration (equal), validation (equal), writing – review and editing (equal). **Meng Shi:** conceptualization (lead), funding acquisition (equal), project administration (equal), supervision (equal), writing – review and editing (equal).

## Conflicts of Interest

The authors declare no conflicts of interest.

## Supporting information


**Figure S1.** Establishment of an animal model of LIRI in mice. (A) Pathological changes in lung tissue (original magnification, ×20). Paraformaldehyde‐fixed sections of lung grafts were stained with HE. (B) The level of BALF protein (C) wet/dry weight ratios (D) capillary permeability and (E) lung injury score in sham and LIRI mice respectively. Data are presented as the means ± standard error of the mean for three independent experiments. ***p < 0.001.
**Figure S2.** ACOX1 and IDH2 protein expression. (A) The representative bands of ACOX1 and IDH2 of Type II AECs in each group. (I) The relative band intensity of ACOX1 and IDH2 in each group. Data are presented as the means ± standard error of the mean for three independent experiments. **p < 0.01.

**Table S1.** List of different metabolites in peripheral serum of LIRI patients vs. healthy participants.

**Table S2.** List of different metabolites in lung tissue of LIRI mice vs. control.

## Data Availability

The datasets used and/or analysed during the current study are available from the corresponding author on reasonable request.
