# Supplementary material for: Succinate Accumulation Accelerates Oxidative Stress to Promote Pulmonary Epithelial Cell Apoptosis During Lung Ischemia–Reperfusion Injury
Source: J Cell Mol Med. 2025 Jun 8;29(11):e70645. doi: 10.1111/jcmm.70645 (PMC12146119; doi:10.1111/jcmm.70645)
Supplement: Supplementary file 1 — Figure S1. Establishment of an animal model of LIRI in mice. (A) Pathological changes in lung tissue (original magnification, ×20). Paraformaldehyde‐fixed sections of lung grafts were stained with HE. (B) The level of BALF protein (C) wet/dry weight ratios (D) capillary permeability and (E) lung injury score in sham and LIRI mice respectively. Data are presented as the means ± standard error of the mean for three independent experiments. ***p < 0.001. Figure S2. ACOX1 and IDH2 protein expression. (A) The representative bands of ACOX1 and IDH2 of Type II AECs in each group. (I) The relative band intensity of ACOX1 and IDH2 in each group. Data are presented as the means ± standard error of the mean for three independent experiments. **p < 0.01. [file JCMM-29-e70645-s003.docx]

**Supplementary Materials for**

**Succinate accumulation accelerates oxidative stress through a mitochondria-dependent pathway during lung ischemia-reperfusion injury**

**Wenhao Wang^1, #^, Nana Feng^2, #^, Qi Shi^3^, Jichun Yang^4^, Yulong Tan^1^, Wenyong Zhou^5, *^, Meng Shi^1, *^**

***Corresponding authors.**

Wenyong Zhou (zhou.wenyong@shsmu.edu.cn) and Meng Shi (mengshi@fudan.edu.cn)

^#^ These authors made equal contributions to this work.

**This file includes:**

Supplementary Figure 1 to Figure 2

**Supplementary Figures and Figure Legends**


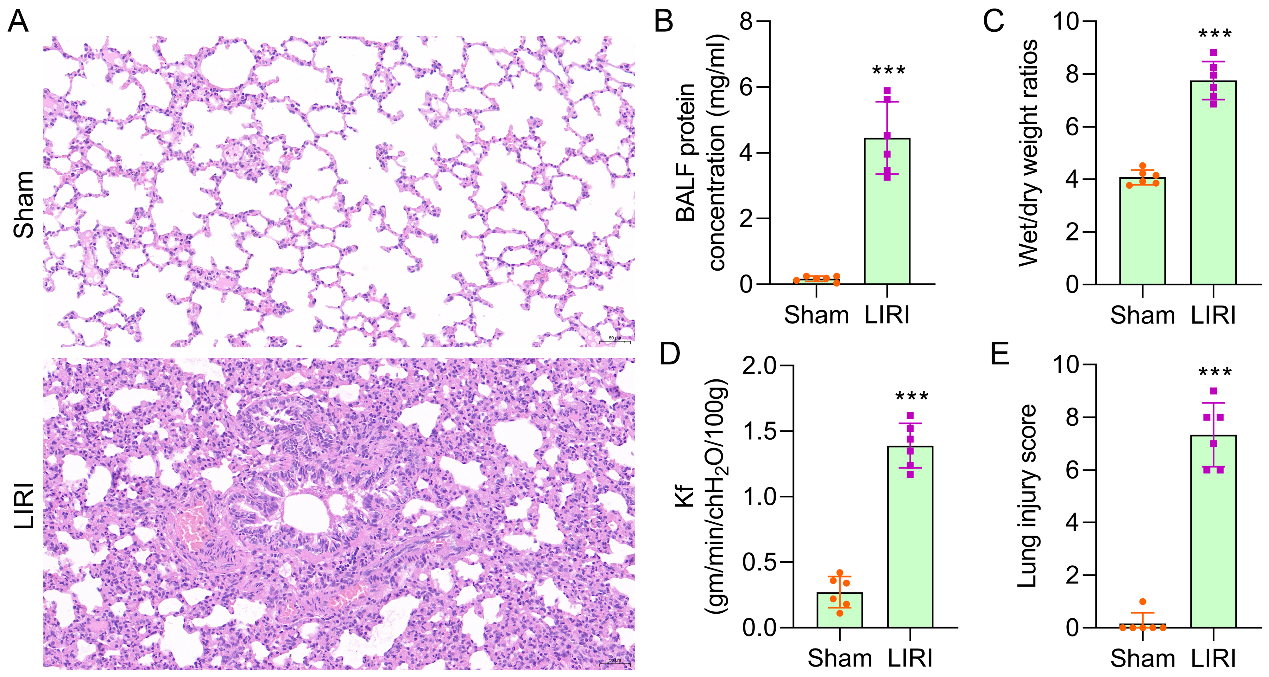


**Figure S1. Establishment of an animal model of LIRI in mice. (A)** Pathological changes in lung tissue (original magnification, ×20). Paraformaldehyde-fixed sections of lung grafts were stained with HE. **(B)** The level of BALF protein **(C)** wet/dry weight ratios **(D)** capillary permeability and **(E)** lung injury score in sham and LIRI mice respectively. Data are presented as the means ± standard error of the mean for three independent experiments. *** *P*＜0.001.


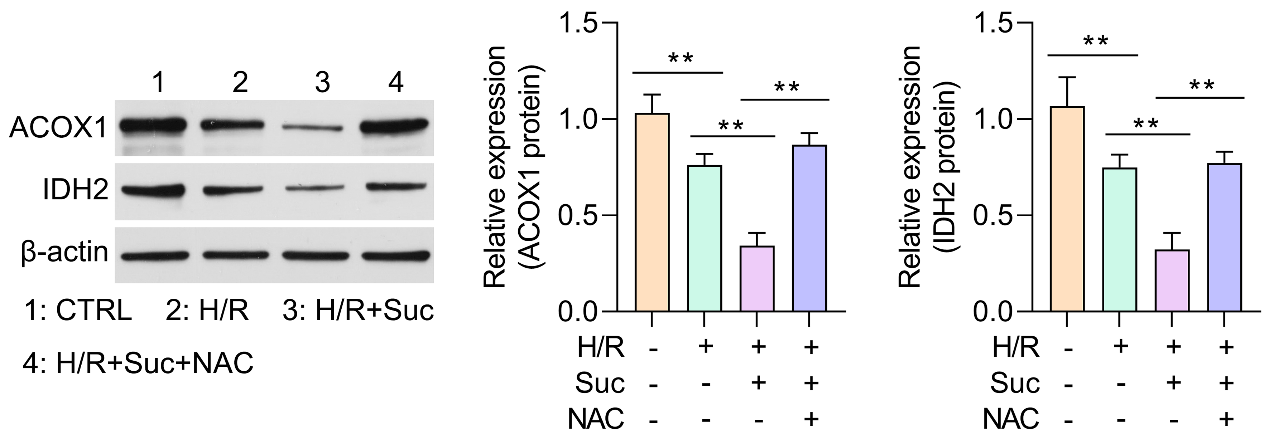


**Figure S2. ACOX1 and IDH2 protein expression. (A)** The representative bands of ACOX1 and IDH2 of Type II AECs in each group. **(I)** The relative band intensity of ACOX1 and IDH2 in each group. Data are presented as the means ± standard error of the mean for three independent experiments. ** *P*＜0.01.
